# Immunohistochemical expression of T-cell subsets (CD4 and CD8) in oral lichen planus

**DOI:** 10.11604/pamj.2023.45.147.38629

**Published:** 2023-08-02

**Authors:** Hiba Jassim Rassol, Taghreed Fadhil Zaidan

**Affiliations:** 1 Department of Oral Diagnosis, College of Dentistry, University of Baghdad, Baghdad, Iraq,; 2 Department of Dentistry, Al-Turath University College, Baghdad, Iraq

**Keywords:** Cluster of differentiation 4, cluster of differentiation 8, oral lichen planus

## Abstract

**Introduction:**

oral lichen planus (OLP) is a common oral mucosal disease with various clinical manifestations. The most predominant types are reticular and erosive. Despite extensive research on the causes of OLP, the exact etiology remains unclear. However, it is believed that a T-cell-mediated response, which triggers the apoptosis of oral epithelial cells, may contribute to the development of this disorder. This study aims to investigate the different types of T-cells (specifically CD4 and CD8) present in OLP tissue samples. By using immunohistochemistry, the expressions of cluster of differentiation 4 (CD4) and cluster of differentiation 8 (CD8) will be evaluated in biopsy samples taken from OLP patients who exhibit various clinical presentations.

**Methods:**

this study was a retrospective analysis study. Oral lichen planus was established histologically in forty paraffin-embedded tissue samples. Blocks of OLP were diagnosed and characterized as reticular or erosive. Immunohistochemical staining was conducted using a monoclonal antibody for (CD4) and a polyclonal antibody for CD8. Semi-quantitative techniques were used to analyze the patterns of positively stained cells. **Results:** forty biopsies of OLP cases were obtained from 24 females and 16 males. The mean age was (49.15±11.39) years. Using an immunohistochemical method, the proportion of CD4 expression: CD8 expression among the epithelial-connective tissue interface was shown to be 24 (60%) cases with a predominance of CD8, 9 (22.5%) cases with no difference, and only 7 (17.5%) cases with a predominance of CD4. The proportion of CD4: CD8 among perivascular parts was shown to be 8 (20%) cases with a predominance of CD8, 20 (50%) cases with no difference, while only 12 (30%) cases had a predominance of CD4. The CD4 perivascular expression was significantly stronger in (71.4%) of erosive OLP than in reticular cases.

**Conclusion:**

T-cell subsets (CD4 and CD8) were found in the OLP infiltrates. The correlation may have contributed to the pathogenesis of OLP.

## Introduction

Oral lichen planus (OLP) is a chronic inflammatory condition in the oral mucosa. Adults of both sexes in middle age are the most frequently affected, with female predilection [1-4]. Anywhere else in the oral cavity may encounter OLP. The gingiva, buccal mucosa, and tongue are often influenced areas, although palatal localization is less frequent. It has various clinical manifestations, such as reticular, papule, plaque-like, atrophic, and ulcerative forms, and may be accompanied by lichen planus of the skin. It is shown to have unique histological, clinical, and characteristic distribution aspects [5]. Oral lichen planus manifests in numerous clinical forms, mainly reticular and erosive. It is histopathologically described as a liquefied basal cell layer with accompanying apoptosis of the keratinocytes, a dense band-like lymphatic infiltrate, and hyperkeratosis [6].

The cause of OLP is unidentified; however, a T-cell-mediated response causing oral epithelial cell apoptosis may be involved [7-10]. The T-cell surface glycoproteins cluster of differentiation 4 (CD4) and a cluster of differentiation 8 (CD8) play a role in T-cell recognition and activation of antigen-presenting cells via interactions with class II and class I major histocompatibility complex (MHC) ligands. There is growing evidence that CD4 and CD8 might chiefly function by complexity with the T-cell receptor to form a 'co-receptor' for identifying antigen-bound MHC molecules, even though they can be engaged with MHC molecules independently of the T-cell receptor [11]. This study aims to investigate the different types of T-cells (specifically CD4 and CD8) present in OLP tissue samples. Using immunohistochemistry, the expressions of CD4 and CD8 will be evaluated in biopsy samples taken from OLP patients exhibiting various clinical presentations.

## Methods

**Study design:** a retrospective cross-sectional study from 2012 to 2020 in the forty confirmed OLP cases using paraffin-embedded tissue blocks and related histopathological reports. Simple random sampling, in which cases are picked purely by chance, is one of the most successful techniques for avoiding sampling bias that researchers may employ. This gives each member of the population an equal chance of being selected to participate in the current study.

**Study samples:** were obtained from the Oral Pathology Laboratory, College of Dentistry, University of Baghdad. Age, sex, and site were extracted from the histopathology report for each OLP case.

**Sample processing:** three sections of 4 μm thickness were cut from each block and placed onto microscopic positively charged slides. One slide was subjected to Hematoxylin and Eosin (H&E) staining to examine the histopathological image of OLP and ensure that an adequate amount of tissue was present. The remaining two slides were subjected to IHC analysis. Immunohistochemically, the procedure includes deparaffinization, rehydration, antigen retrieval, blocking endogenous peroxidase, and adding primary and secondary antibodies to the slides. A monoclonal anti-CD4 antibody (E-AB-22098, diluted 1: 200) and a polyclonal anti-CD8 antibody (E-AB-19675, diluted 1: 100) from Elabscience Biotechnology, Wuhan, China, were utilized as primary antibodies in this investigation.

**The scoring system:** using a light microscope at 400X magnification, two pathologists independently examined five random fields from each section to determine Immunohistochemistry (IHC) expression. A four-step grade was utilized to assess the immunopositivity of each antigen (CD4 and CD8): negative, mild, moderate, and strong. Positive cells were categorized as negative (0%), mild (0-10%), moderate (10-50%), and strong (50-100%). The ratio of CD4-positive cells to CD8-positive cells was compared using a method that had been adjusted by Tímár *et al*. [12]. The antigen with wider reactivity (predominate) was given a 3 (strong). The antigen with less reactivity (a lower proportion of cells) was given a 1 (weak) or 2 (moderate) value, depending on the difference in reactivity [12,13]. As a result, the CD4+: CD8+ ratio (<1, with CD8+ T- T-cells predominating), while the CD4+: CD8+ ratio (>1, with CD4+ T-cells predominating) [12].

**Sample size determination and sampling technique:** sample size was determined using a single population proportion formula by considering the prevalence of OLP ranges from 0.2% to 4% proportion according to previous studies [5,6,14]. The formula for calculating the sample size (n) is as follows [15]:


$$N=\frac{Z^{2}p\left(1-p\right)}{d^{2}}$$

Where N: sample size; Z: is the standard deviation at a confidence level of 95%, which is 1.96. P: is the estimated percentage of probability for the event to be measured. 1-p: is the probability for the event not to occur; d: is the percentage of the acceptable error, which is in our study equal to 0.05.


$$N=\frac{1.96^{2}x0.02\left(1-0.02\right)}{0.05^{2}}=30$$

A 5% of the calculated sample size was added for the potential non-response, then became 38, which was approximated for 40 subjects.

**Statistical analysis:** a statistical package for the social sciences (SPSS version 23) was used to analyze the data. Descriptive statistics were presented as frequency tables; Continuous variables were expressed as mean ± standard deviation and categorical variables as numbers and percentages. Analytic statistics, such as the Chi-square test, were used to estimate the association between two categorical variables. The P-value < 0.05 was regarded as significant.

**Ethical approval:** this study was performed after receiving ethical approval from the Ethics Committee, College of Dentistry, University of Baghdad (Ref # 272, date: March 25, 2021).

## Results

**Figure 1:**
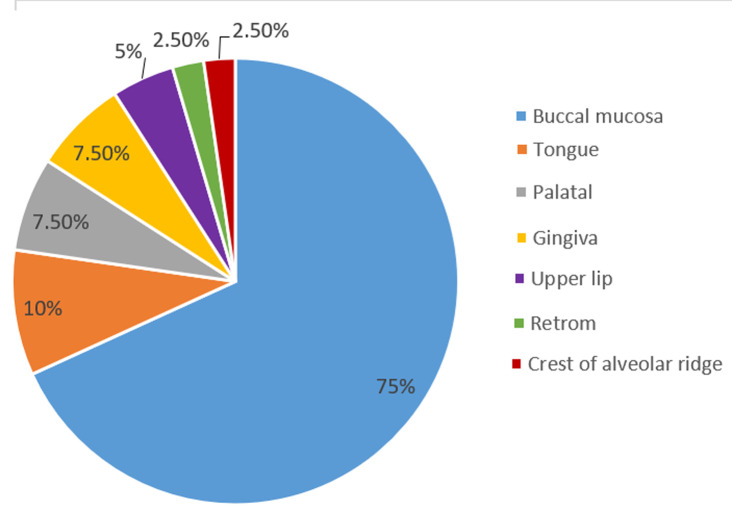
sites of lichen planus in the oral cavity

In this study, forty cases of OLP were examined. The mean ±SD of age was 49.15 ± 11.39 years, ranging between 28-69 years, with a male-to-female ratio of 1: 1.5. The age and sex distribution for the studied group is shown in Table 1. The clinical presentation of the studied cases showed that 21 (52.5%) cases had the erosive type of lichen planus, and 19 (47.5%) cases had the reticular type (Table 2). Regarding the observed distribution of sites, about two-thirds of the studied cases with OLP were present in the buccal mucosa site, accounting for 30 (75% of the cases). In contrast, the rest of the other cases were distributed in small numbers, as shown in Figure 1.

**Table 1:** demographic findings of studied cases

Demographic character		Number	Percentage	
**Age**	<40 years	7	17.5%	
	40-49 years	13	32.5%	
	50-59 years	10	25.0%	
	≥60 years	10	25.0%	
**Mean ± SD range**		49.15 ± 11.39 (28-69)		
**Gender**	Male	16	40.0%	Mean ± SD (45.81 ± 10.41)
	Female	24	60.0%	Mean ± SD (51.37 ± 11.68)
**Total**		40	100.0%	

**Table 2:** clinical types of oral lichen planus among the studied group

Clinical presentation		Number	Percentage
**Clinical type**	Erosive	21	52.5%
	Reticular	19	47.5%
**Total**		40	100%

**Figure 2:**
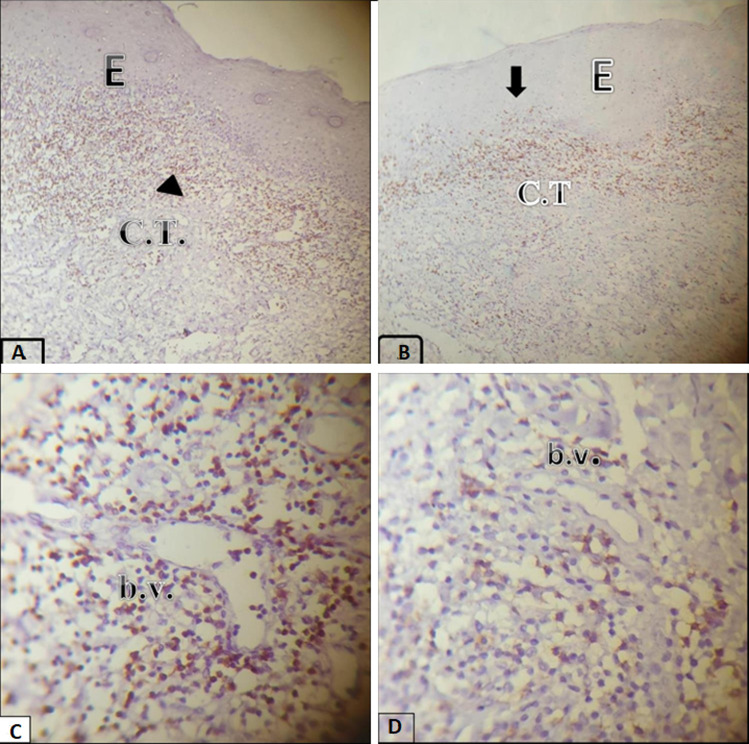
immunohistochemical expression of (CD4+) and (CD8+) markers for the same section of OLP; (A) the majority of CD4+ expression found in the connective tissue underlying the involved epithelium (lamina propria) (head arrow) at (×100); (B) the CD8marker expression localized in ECTI with clusters of intraepithelial CD8 T-cells (arrow) at (×100); (C) the CD4+ marker expression showing CD4-positive cells in the perivascular site at (×400) with camera magnification; (D) the CD8+ marker expression showing CD8-positive cells in the perivascular location (×400)

**Figure 3:**
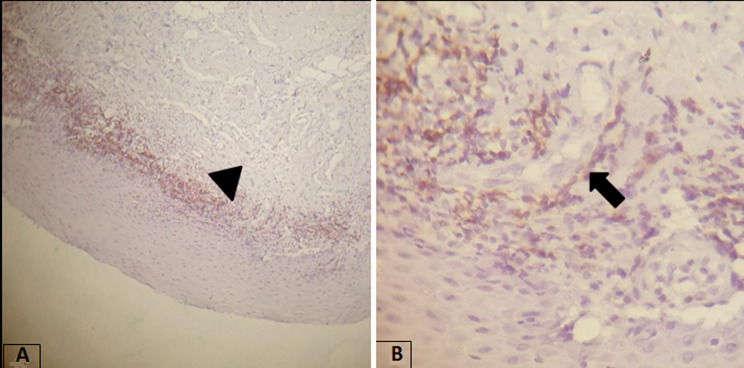
immunohistochemical expression of CD4+ marker in OLP cases; (A) the CD4 marker expression predominated in the perivascular site (head arrow) (×100); (B) high-power view of the previous section (×400)

**Figure 4:**
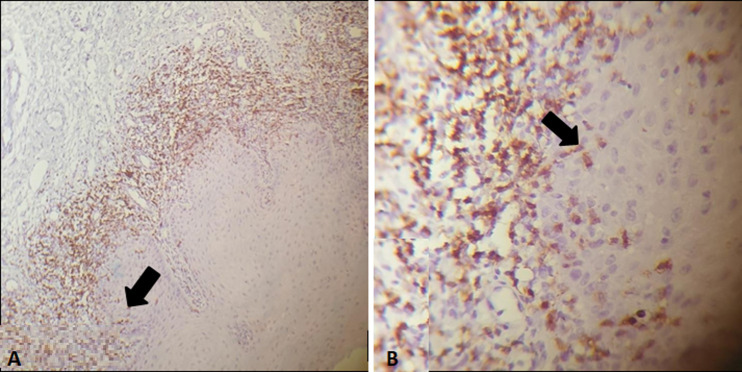
immunohistochemical expression of CD8+marker in OLP cases; (A) the CD8 marker expression dominated in the perivascular site and the ECTI, with intraepithelial CD8 T-cell clusters (arrow) at (×100); (B) high-power view of the previous section (×400)

**Evaluation of IHC expression of T-cell subsets markers (CD4 and CD8) in OLP cases:** the T-cell subset markers (CD4 and CD8) expressions were found at two different places: at the epithelial-connective tissue interface (ECTI) and in the perivascular site. It was shown that 12 (30%) cases had strong CD4 T-cell subset marker expression in the ECTI, while 22 (55%) cases had strong CD4 expression in the perivascular site. And 24 (60%) cases had strong CD8 in the ECTI, while 16 (40%) cases had strong CD8 expression in the perivascular site (Table 3, Table 4) and (Figure 2, Figure 3, Figure 4). The proportion of CD4: CD8 among ECTI and perivascular sites is shown in (Table 4).

**Table 3:** expression of T-cell subsets markers among studied cases

CD4 and CD8 immunohistochemistry		T-cell marker	Number	Percentage
**CD4**	ECTI	Mild	15	37.5%
		Moderate	13	32.5%
		Strong	12	30.0%
	Perivascular site	None	2	5.0%
		Mild	8	20.0%
		Moderate	8	20.0%
		Strong	22	55.0%
**CD8**	ECTI	Moderate	16	40.0%
		Strong	24	60.0%
	Perivascular site	Mild	12	30.0%
		Moderate	12	30.0%
		Strong	16	40.0%
**Total**			40	100%

CD4: cluster of differentiation 4, CD8: cluster of differentiation 8, ECTI: epithelial-connective tissue interface

**Table 4:** proportion of CD4:CD8 among ECTI and perivascular sites

Proportion of CD4: CD8			Number	Percentage
**ECTI CD4: CD8**		Predominance CD8	24	60.0%
		No difference	9	22.5%
		Predominance CD4	7	17.5%
**Perivascular CD4: CD8**	**Site**	Predominance CD8	8	20.0%
		No difference	20	50.0%
		Predominance CD4	12	30.0%

CD4: cluster of differentiation 4, CD8: cluster of differentiation 8, ECTI: epithelial-connective tissue interface

**The expression of CD4 and CD8 at ECTI and perivascular sites according to clinical types of OLP:** the epithelial-connective tissue interface CD4 and CD8 expression showed no significant differences according to clinical types (p>0.05). Despite strong CD4 and CD8 expression in 8 (38.1%) and 13 (61.9) ECTI sites of erosive OLP cases, which were higher than those in reticular OLP cases, but showed no significant differences between both types of OLP p>0.05 (Table 5). The perivascular CD4 expression was significantly associated with clinical types (P = 0.028), whereas the erosive type had strong perivascular CD4 expression in 15 (71.4%) cases. The reticular type had strong perivascular CD4 expression in only 7 (36.8%) cases. Perivascular CD8 expression was not significantly associated with the clinical types of the examined groups (p>0.05). However, strong CD8 expression in samples with erosive clinical type 10 (47.6%) was higher than in samples with reticular clinical type 6 (31.6%) (Table 6).

**Table 5:** the ECTI CD4 and CD8 expressions according to clinical types of studied cases of OLP

Variable		ECTI CD4 expression			Chi-square	P-value
**Mild**	**Moderate**	**Strong**
Clinical types	Erosive	7(33.3%)	6(28.6%)	8(38.1%)	1.38	0.501; NS
	Reticular	8(42.1%)	7(36.8%)	4(21.1%)		
**Variable**		**ECTI CD8 expression**			**Chi-square**	**P-value**
		**Moderate**	**Strong**			
Clinical types	Erosive	8(38.1%)	13(61.9%)		0.067	0.79; NS
	Reticular	8(42.1%)	11(57.9%)			

NS: No significant; CD4: cluster of differentiation 4, CD8: cluster of differentiation 8, ECTI: epithelial-connective tissue interface, OLP: oral lichen planus

**Table 6:** the perivascular CD4 and CD8 expression according to clinical types of studied cases of OLP

Variable		Perivascular CD4 expression				Chi-square	P-value
		Negative	Mild	Moderate	Strong		
Clinical type	Erosive	1(4.8%)	1(4.8%)	4(19%)	15(71.4%)	4.82	0.028*
	Reticular	1(5.3%)	7(36.8%)	4(21.1%)	7(36.8%)		
**Variable**		**Perivascular CD8 expression**				**Chi-square**	**P-value**
		**Mild**	**Moderate**	**Strong**			
Clinical type	Erosive	4(19%)	7(33.3%)	10(47.6%)		2.57	0.27
	Reticular	8(42.1%)	5(26.3%)	6(31.6%)			

* Significant p<0.05, CD4: cluster of differentiation 4, CD8: cluster of differentiation 8, OLP: oral lichen planus

## Discussion

The age range of patients with OLP in this study was from 28 to 69 years old, with a mean age of (49.15±11.39). The results of prior epidemiological investigations showed a wide variety of median ages. Generally, age, sex, and clinical presentation are all essential considerations regarding epidemiological parameters. Due to the limited number of cases and type of research, it may be held responsible for inconsistent findings in the current study and several other, not epidemiological ones [1,4].

**The IHC expression of T-cell subsets markers (CD4 and CD8):** the immunohistochemical expression of T-cell subset markers (CD4 and CD8) showed positive immunoreactivity in the ECTI and the perivascular site, revealing that 12 (30%) cases had strong CD4 T-cell subset marker expression in the ECTI, 13 (32.5%) cases had a moderate expression, 15 (37.5%) cases had a mild expression, and 22 (55%) cases had strong CD4 expression in the perivascular site. And 24 (60%) cases had strong CD8 expression in the ECTI, while 16 (40%) cases had strong CD8 expression in the perivascular site. The proportion of CD4: CD8 among the ECTI showed that 24 (60%) cases had a predominance of CD8, 9 (22.5%) cases had no difference, and only 7 (17.5%) cases had a predominance of CD4. The proportion of CD4: CD8 among perivascular parts showed that 5 (20%) cases had a predominance of CD8, 20 (50%) cases had no difference, and 12 (30%) cases had a predominance of CD4.

So the infiltrates of keratinocytes in the basal layer of epithelium in 60% of studied cases of OLP were predominantly CD8+ suppressor-cytotoxic cells; this is consistent with [16-20], who demonstrated that CD8+ T-cells make up most of OLP's cellular infiltration relative to CD4+ T-cells, elucidating the pathogenic involvement of CD8+ T-cells in basement membrane destruction, lends credence to the idea that cytotoxic T lymphocytes have a role in the disease's emergence. Whereas others have demonstrated that CD4+ T-cells (helper cells) were more predominant than CD8+ T-cells (cytotoxic cells) in the infiltrate, as was shown in 7 (17.5%) of studied cases of OLP, this was by [13,21-24] who suggested that T helper cells CD4+ are abundant in the early lesion. Early lesions may have more CD4 cells, which promotes the entry of CD8 cells observed in advanced lesions. As mentioned earlier, CD 4 T-cells in this study showed in 55% of cases had strong CD4 in the perivascular site that was more prominent than CD8 cells in lamina propria, in particular at the perivascular site in 30% of studied cases, this was in agreement with [13,24,25]. Regarding the proportion of CD4: CD8 cells present in the perivascular site, CD4 positive T-cells predominated over CD8-positive T-cells in 12 (30%) of the OLP studied cases; this was agreed with [13,24,25]; who concluded that CD4 T-cells were more prominent than CD8 cells in the lamina propria, particularly in the perivascular site. The perivascular distribution of CD4+ T-cells indicates the potential help from Th1 cytokines to cytotoxic CD8+ cells. It is uncertain how T-cells are attracted to OLP tissues, although OLP has been shown to exhibit T-cell characteristics. Al-Drobie [26] showed that lymphocyte emigration occurs through subepithelial blood vessels. Zhu *et al*. [27] stated that CD4 T-cells could be crucial in stimulating the recruitment and/or entrance of CD8 T-cells into the epithelium. Rana *et al*. [28] detected that CD4+ T-cells predict potential Th1 cytokine-mediated support for cytotoxic CD8+ cells. If CD8+ T-cells killed keratinocytes, the basement membrane would break down. This would let more CD8+ T-cells in, killing more basal cells and keeping the vicious cycle that keeps chronic diseases going.

**The expression of CD4 and CD8 (at ECTI and perivascular site) according to clinical types of OLP:** as far as clinical types of OLP cases (erosive and reticular). The results showed that at ECTI, the CD4 marker was strongly expressed in 4 (21.1%) cases of the reticular type and 8 (38.1%) cases of the erosive type. On the other hand, the CD8 marker was strongly expressed in 11 (57.9%) cases of reticular OLP and 13 (61.9%) cases of erosive OLP. The difference, however, was not statistically significant (p > 0.05). At the perivascular site, the CD4 marker was strongly expressed in 7 (34.8%) cases of the reticular type and 15 (71.4%) cases of the erosive type. There was a significant association between clinical type and perivascular CD4 (P = 0.028). On the other hand, the CD8 T-cell marker was strongly expressed in 6 (31.6%) cases of reticular OLP and 10 (47.6%) of the erosive OLP cases, but the difference was not statistically significant (p > 0.05). So concerning clinical types, it was observed that CD4 and CD8 markers had stronger expression in erosive type OLP than reticular type OLP. These results agree with Jana *et al*. [29], who found that the OLP-reticular type is less severe in terms of inflammation than the erosive form and confirm a statistically significant association between the clinical type and the perivascular site CD4 expression, in which the erosive type had a strong expression for CD4 in 15 (71.4%) cases, while the reticular type had a strong CD4 in only 7 (36.8%) cases. This study is comparable to that of Wang *et al*. [30], who found that the CD4+ T helper subset may play a prominent role in OLP immunopathology and demonstrated that tissue inflammation is orchestrated by proinflammatory cytokines (IL-6, IL-1, and TNF) and chemokines via CD4 (Th17) that attract and activate Th1 cells. The CD4+ T helper subset cell (Th17) might play an essential role in erosive OLP and might be to blame for more obvious oral mucosal destruction.

This study reveals that the markers above were positively expressed in most of the studied cases, suggesting that each may independently influence the OLP's evolution The present study has some limitations. First, there are no direct clinical examinations and the data depended on the histopathological reports. Second, this study has a small sample size; therefore, these results must be substantiated in a more significant sample of OLP cases.

## Conclusion

This study revealed that (CD4 and CD8 T-cells) were positively expressed in most of the studied cases, which suggests that each of them may act independently of the other in influencing the OLP's emergence. In cases of erosive OLP, CD4 perivascular expression was significantly higher than in cases of reticular OLP.

### What is known about this topic

- *Oral lichen planus is a T-cell-mediated immune response inducing oral epithelial cell apoptosis that may cause the disorder;*
- *Both helper T-cells and cytotoxic T-cells were found in the OLP infiltrates*.

### What this study adds

- *The dominance of intraepithelial CD8+ T-cells reactivity and their distribution in the basal cell layer adjacent to an area of cell damage in OLP lends credence to the idea that cytotoxic T lymphocytes have a role in the disease's emergence;*
- *It was observed that CD4 and CD8 markers had stronger expression in erosive type OLP than reticular type OLP*.
